# Pt/Ru-Modified ZnO Sensor Grown In Situ for Detection of ppb H_2_S

**DOI:** 10.3390/s25071995

**Published:** 2025-03-22

**Authors:** Jianhua Zhang, Yunbo Shi, Bolun Tang, Canda Zheng

**Affiliations:** Higher Educational Key Laboratory for Measuring & Control Technology and Instrumentations of Heilongjiang Province, Harbin University of Science and Technology, Harbin 150080, China; zhangjianhua0919@163.com (J.Z.); tangbolun@sina.com (B.T.); zhengcanda@outlook.com (C.Z.)

**Keywords:** gas sensors, ZnO-Pt/Ru, H_2_S detection, low detection limit, air quality

## Abstract

This paper presents a ZnO-Pt/Ru sensor prepared by a two-step hydrothermal method with in situ-grown ZnO nanorods and doped with Pt and Ru elements by immersion sintering. Characterization results showed that Pt and Ru were successfully modified on the surface of ZnO nanorods. ZnO-Pt/Ru achieved a response of 25–50 ppm H_2_S at the optimum operating temperature of 198 °C. In addition, the lower limit of H_2_S detection of ZnO-Pt/Ru reached 50 ppb with a response of about 10%, indicating a wide concentration detection range. Due to the good catalytic properties of Pt, the recovery characteristics of ZnO at high concentrations of H_2_S were significantly improved. The response time of ZnO-Pt/Ru (30 s) was also significantly shorter than pristine ZnO (56 s), with excellent selectivity. As far as the gas-sensitive enhancement mechanism is concerned, at the macroscopic level, the ZnO surface was modified by Pt and Ru, and this special structure of ZnO-Pt/Ru significantly increased the specific surface area. At the microscopic level, the PN junction formed between Pt/Ru and ZnO provided abundant holes for electron migration.

## 1. Introduction

Hydrogen sulfide (H_2_S) is a highly toxic, corrosive, and flammable gas with a rotten egg odor. It is typically derived from organic matter such as crude oil, natural gas, food, and the bacterial decomposition of human and animal waste. Exposure to high concentrations of H_2_S may cause physical discomfort, including a sore throat, coughing, eye irritation, etc. At concentrations as high as 1000 ppm, it can even cause immediate death [1].

Currently, the main methods for detecting H_2_S include chemical resistance and gas sensitivity, electrochemical detection based on redox reactions, gas chromatography, and optical methods [2]. However, most of these detection techniques typically have drawbacks such as complex structures and high costs [3]. For example, gas chromatography is unsuitable for on-site monitoring due to its complicated operation process and expensive and large equipment [4]. In recent years, gas sensors based on metal oxide semiconductors (MOs) have attracted significant attention due to their high response, low cost, and other advantages [5,6].

Typical metal oxides include CuO, WO_3_, ZnO, and SnO_2_, all widely studied for H_2_S gas-sensing detection. ZnO, as an N-type semiconductor, is characterized by a wide bandgap energy of approximately 3.3 eV. It also has advantages such as high electron mobility, non-toxicity, thermal stability, and chemical stability. The fabrication and testing of H_2_S sensors based on various ZnO nanostructures has been widely studied.

For example, Cu-doped ZnO films were deposited on glass substrates by Shewale et al. using spray pyrolysis technology, and the response to 20 ppm H_2_S at 260 °C reached 0.38 [7]. Xuan et al. prepared a ZnO nanoparticle sensor in situ on a gas-sensitive electrode through a one-step simple sol-gel method, which had a response of 0.75–750 ppb H_2_S at room temperature [8]. Niyanta et al. successfully fabricated CuO-coated ZnO nanowires on Si/SiO_2_ substrates via a hydrothermal method using thermal evaporation, which had a high response of 40–10 ppm H_2_S at 200 °C [9]. A flower-like hierarchical ZnO structure, characterized by rapid response, was synthesized by Fan et al. through a combination of electrospinning and hydrothermal methods [10]. Wu et al. prepared MOF ZIF-8/ZnO composite structures via the template method with a detection limit as low as 50 ppb, showing excellent selectivity to H_2_S compared to other reducing gases [11].

Although MOs have been widely applied in gas-sensing detection, their sensing performance still requires improvement. One of the ways to improve sensor performance is the modification of the MO surface using metal sensitizers such as Pt, Ru, Pd, Ag, etc. For example, Kruefu et al. studied Ru-loaded WO_3_ synthesized via a hydrothermal/immersion method, which exhibited excellent selectivity and response capabilities toward H_2_S [12]. ZnO nanorods were functionalized and modified with Pt by Yu et al. using a simple immersion-calcination method, achieving a lower detection limit [13]. Su et al. showed that Pd functionalization significantly reduced the operating temperature and increased the operating range compared to SnO_2_ [14].

Bimetals consist of two precious metals: Pt/Pd, Ag/Pt, and Au/Pd. Compared to single metals, bimetals typically exhibit superior gas-sensing properties due to the synergistic effects between components. For example, Pd/Pt nanoparticles were uniformly modified on networked SnO_2_ nanowires by Choi et al. using sequential radiative analysis, which reduced the response and recovery times of the sensor for NO_2_ [15]. Chen et al. used the wet impregnation method to load Pt and Ru onto the surface of WO_3_ nanoparticles prepared by tungstic acid pyrolysis to enhance the sensitivity of WO_3_ nanowire sensors to acetone [16]. Pd/Au-loaded ZnO nanowires were fabricated by Shen et al. using a simple mixed solution heating evaporation method, improving the selectivity and reproducibility for NO_2_ [17]. Pd/Pt bimetal-modified In_2_O_3_ hollow spheres were synthesized by Jiang et al. using solvothermal and in situ reduction methods, enabling trace H_2_S detection with excellent selectivity and anti-interference capabilities [18].

Platinum and ruthenium are both noble metals with excellent catalytic properties and potential synergies between them. Therefore, a bimetallic Pt and Ru-modified ZnO gas sensor for H_2_S detection was fabricated via a two-step hydrothermal growth method in this study. The structure and morphology of the composite material were analyzed using scanning electron microscopy (SEM), X-ray diffraction (XRD), X-ray photoelectron spectroscopy (XPS), and transmission electron microscopy (TEM). Currently, there are few studies on Pt and Ru as co-doped materials, and the in situ hydrothermal growth method is different from traditional coatings. It also promotes the connection between doped elements and ZnO while increasing the specific surface area of ZnO nanorods. Subsequent gas-sensing tests showed that the sensor showed a high response (15 ppm/790%) at high concentrations and a very low detection limit (50 ppb), with a wide concentration detection range, and demonstrated good recovery and rapid response performance. These results were attributed to chemical sensitization and electronic sensitization mechanisms.

## 2. Materials and Methods

### 2.1. Preparation of Sensing Materials

Chemicals: Zn (CH_3_COO)_2_·2H_2_O (CAS number 5970-45-6, 99%), C_6_H_12_N_4_ (CAS number 100-97-0, 99%), Zn (NO_3_)_2_·6H_2_O (CAS number 10196-18-6, 99%), anhydrous ethanol (CAS number 64-17-5, >99.7%), H_2_PtCl_6_ (CAS number 18497-13-7, analytical grade, 37%), and RuCl_3_ (CAS number 14898-67-0, 98%).

All chemicals were purchased from Shanghai McLin Biochemical Technology Co., Ltd. (Shanghai, China) and Shanghai Aladdin Biochemical Technology Co., Ltd. (Shanghai, China).

### 2.2. Synthesis of ZnO-Pt/Ru Nanorod

ZnO seed layer preparation: First, the alumina substrates were immersed in acetone, anhydrous ethanol, and deionized water, respectively, and ultrasonically cleaned for 10 min. The alumina substrates were immersed in a 0.01 mol/L Zn (CH_3_COO)_2_·2H_2_O ethanol mixed solution for 4 min, then placed in a vacuum drying oven (DZF6090, Changzhou Henglong Instrument Co., Changzhou, China) at 80 °C for 5 min of annealing, repeated four times. Finally, the alumina substrates were heated in a muffle furnace (SGM28, Luoyang Sigma High-Temperature Furnace Co., Luoyang, China) at a rate of 2 °C/min to 400 °C, where they were kept for 2 h and then cooled naturally to room temperature.

Preparation of ZnO Growth Precursor Solution: 0.2102 g of C_6_H_12_N_4_ and 0.4464 g of Zn (NO_3_)_2_·6H_2_O were dissolved in 50 mL of deionized water, and the concentration was 30 mmol/L. The pre-sintered alumina substrates and the precursor solution were placed in a Teflon-lined hydrothermal autoclave and heated at 95 °C for 3 h. After natural cooling, the substrates were removed and washed with deionized water and anhydrous ethanol three times, respectively. The cleaned alumina substrates were aged at a DC voltage of 5 V for 3 days, and then used as the original ZnO sensor.

Pt/Ru doping: An ethanol solution of 0.06 M H_2_PtCl_6_ and 0.03 M RuCl_3_ was prepared. The original alumina substrates with ZnO nanorods were immersed in the two solutions for 10 min, followed by calcination at 500 °C for 3 h. In this way, ZnO nanorods doped with Pt and Ru were obtained (ZnO-Ru, ZnO-Pt).

**Figure 1:**
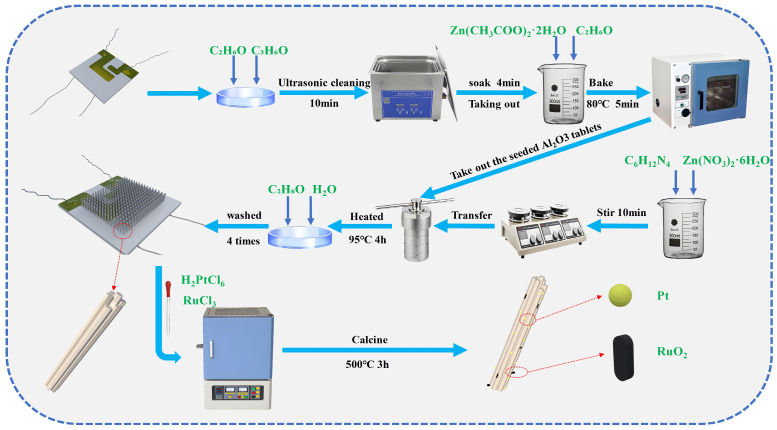
Schematic of the fabrication process for ZnO-Pt/Ru and other gas sensors.

A total of 0.06 M H_2_PtCl_6_, 0.03 M RuCl_3_, and anhydrous ethanol were added to a beaker. The mixture was stirred on a magnetic stirrer for 10 min. The remaining preparation process was the same as described above. Thus, ZnO doped with both Pt and Ru (ZnO-Pt/Ru) was successfully prepared. The overall preparation process is shown in Figure 1. Regarding the selection of Pt and Ru doping amounts, a review of relevant literature indicated that excessive doping decreases sensing performance. After comprehensive consideration, Pt doping was set at 0.5 wt% [19,20,21] and Ru doping at 0.25 wt% [22,23,24].

### 2.3. Characterization of Sensing Materials

The crystal structure of the samples was analyzed using X-ray diffraction (XRD, Rigaku Smart Lab, Rigaku Corporation, Tokyo, Japan, Cu Kα radiation, λ = 1.5406 Å) in the 2θ range of 10–80°. The surface morphology and elemental composition of the samples were observed using a field-emission scanning electron microscope (SEM, TESCAN MIRA LMS, Tescan Co., Ltd., Brno, Czech Republic; Zeiss Sigma 500, Oberkochen, Germany) with an energy-dispersive X-ray spectroscopy (EDS) detector, operating at an acceleration voltage of 15 kV and 3 kV, respectively. The valence states of each element and the chemical composition of the samples were analyzed using X-ray photoelectron spectroscopy (XPS, Thermo Scientific K-Alpha XPS system, Thermo Fisher Scientific, Waltham, MA, USA) with Al Kα radiation. The specific surface area and pore size of the samples were determined using a porosity analyzer (Micromeritics 3Flex, Norcross, GA, USA), and the nitrogen adsorption/desorption isotherms were determined at 468 K. The microstructure and crystallinity of the samples were further investigated by transmission electron microscopy (TEM, JEM-F200, JEOL Ltd., Tokyo, Japan) and energy-dispersive spectroscopy (EDS, JEM-F200, JEOL Ltd., Tokyo, Japan).

### 2.4. Gas-Sensing Testing Environment

The test environment consisted of a 10 L sealed gas chamber, a data acquisition card, and a computer terminal. The data acquisition card was used to collect the resistance value of the sensor in the air and gas to be tested. The test method used static measurements, where the concentration of the target gas in the gas chamber was controlled by introducing a quantitative volume of the target gas into an air-filled 10 L gas chamber. For example, to measure a 1 ppm gas, if a 1000 ppm standard gas is used as the gas source, 10 mL of the gas source must be injected into the gas chamber. During the test, the response value was calculated according to the formula (Ra − Rg)/Rg. In gas-sensitive characterization, the response time is defined as T_90_: the time required for the resistance to change by 90% from the start of the response to the end of the response. The recovery time is defined as RT_90_: the time required for the resistance to change by 90% from the time of removal from the target gas environment.

## 3. Results and Discussion

### 3.1. Characterization of Material

**Figure 2:**
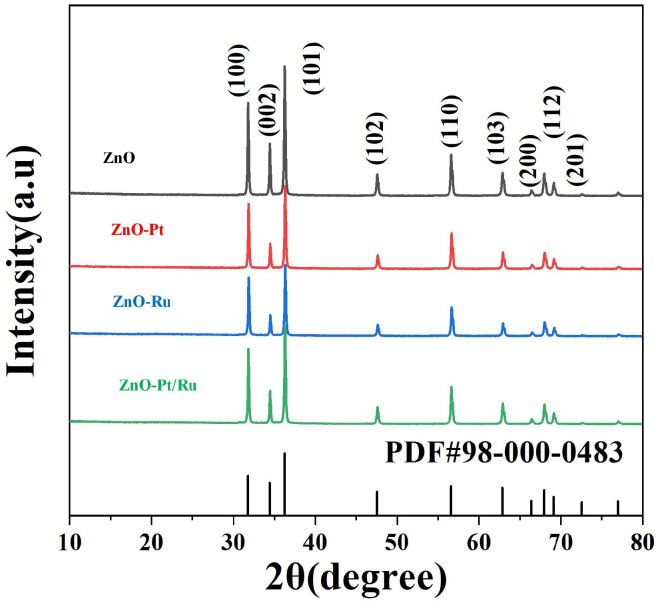
X-ray diffraction patterns of pure ZnO, ZnO-Ru, ZnO-Pt, and ZnO-Pt/Ru.

Figure 2 shows the XRD spectra of the original ZnO, ZnO-Ru, ZnO-Pt, ZnO-Pt/Ru, and ZnO standard PDF card (PDF#98-000-0483). The main peaks of all four materials corresponded to the (101), (100), and (002) of ZnO. It was observed that ZnO predominantly grows along the (101) direction in all samples.

**Figure 3:**
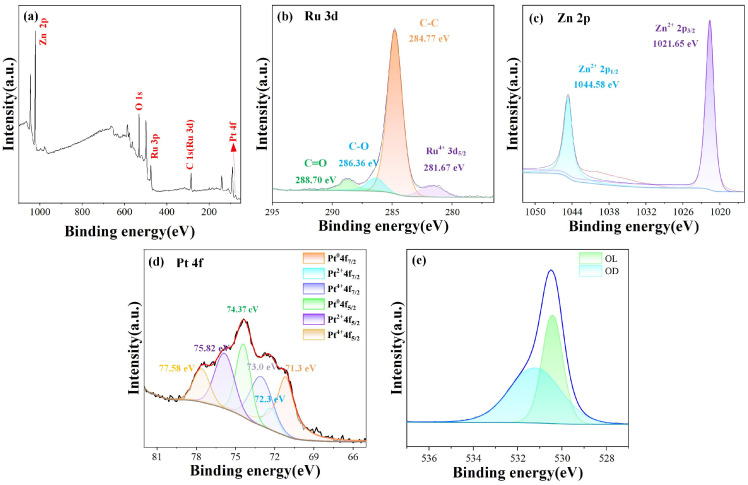
XPS spectra of ZnO-Pt/Ru: (**a**) Survey; (**b**) Ru 3d; (**c**) Zn 2p; (**d**) Pt 4f; (**e**) O1s.

XPS was used to detect the elemental composition and chemical states of the materials. As shown in the full spectrum in Figure 3a, Pt and Ru were detected in the sample. The C1s peak at 284.8 eV was used as a baseline for calibration. Figure 3b shows the main peak of C1s at 284.8 eV. After convolution fitting, three peaks were observed at 284.76 eV, 286.36 eV, and 288.7 eV, corresponding to C-C, C-O, and C=O, respectively.

Additionally, because the 3d orbitals of Ru overlap significantly with the C1s peak, peak deconvolution was performed. The peak located at 281.67 eV corresponds to the 3d^5/2^ state of Ru [25]. Figure 3c shows the Zn 2p spectrum of the ZnO-Pt/Ru composites. The Zn 2p orbitals were spin-split into Zn 2p^1/2^ and Zn 2p^3/2^, with binding energies of 1021.65 eV and 1044.58 eV. The double peak distance was 22.93 eV, indicating the presence of Zn^2+^ and confirming the formation of ZnO [26]. Figure 3d shows the Pt 4f spectrum, which can be deconvoluted into three pairs of peaks [27,28]. The Pt 4f 7/2 and 4f 5/2 peaks at 71.1 and 74.4 eV correspond to Pt^0+^, while the peaks at 72.2 eV and 75.8 eV indicate the formation of Pt^2+^. The fitted peaks at 73.0 and 77.6 eV correspond to Pt^4+^, indicating the presence of Pt and its oxide, PtO_x_. The O1s spectrum shown in Figure 3e is deconvoluted into two peaks at 530.4 eV and 531.2 eV, which was attributed to lattice oxygen and adsorbed oxygen, respectively [29].

**Figure 4:**
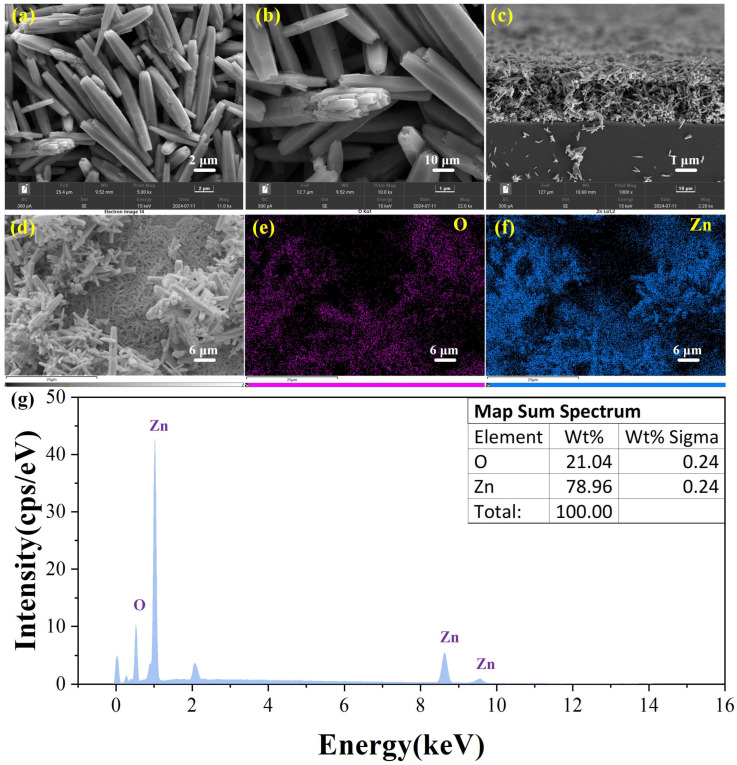
SEM images of (**a**,**b**) ZnO with different magnifications; cross-sectional side view under the silicon substrate of (**c**) pure ZnO; EDS elemental mapping profiles of (**e**) O and (**f**) Zn in pure ZnO from (**d**); (**g**) EDS spectrum of ZnO.

Figure 4a shows the SEM image of ZnO where the ZnO nanorods of ZnO nanorods are mainly concentrated at 747 ± 356 nm in diameter and 5.849 ± 2.713 um in length. The cross-section exhibits a unique hexagonal structure. The cross-stacked nanorods increase the contact area with the target gas and enhance the adsorption and desorption of gases. Figure 4b is a partially enlarged view of a single rod. It can be observed that the structure inside a single nanorod consists of multiple smaller nanorods. Figure 4c shows that the nanorods with a length of 4–6 µm are cross-arranged and form a relatively regular ZnO film on the substrate. Compared to traditional coating methods, this approach increases the voids, allowing more space for gas interaction with the material. Figure 4e–g show the EDS spectra of ZnO. It can be observed that the distribution of Zn and O is roughly consistent with the distribution of ZnO nanorods in the images and there are no impurity elements.

**Figure 5:**
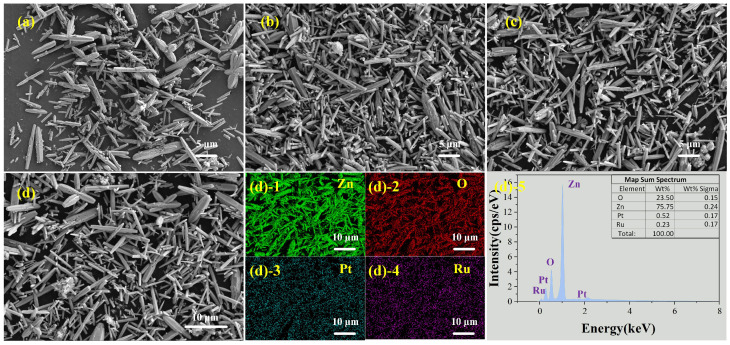
SEM images of (**a**) ZnO-Pt, (**b**) ZnO-Ru and (**c**) ZnO-Pt/Ru; EDS analysis of ((**d)-1**–**(d)-5**) from (**d**).

Figure 5a–c shows SEM images of ZnO-Pt, ZnO-Ru, and ZnO-Pt/Ru respectively. Figure 5d shows the EDS pattern of ZnO-Pt/Ru. As shown in Figure 5d, Pt and Ru in ZnO-Pt/Ru are successfully doped with an expected doping ratio of 0.52 wt% and 0.23 wt%, respectively. Although Pt and Ru are successfully doped, the material structure that distinguishes ZnO nanorods is not clearly observed in Figure 5a–c. To investigate further, we used TEM to characterize ZnO and ZnO-Pt/Ru.

**Figure 6:**
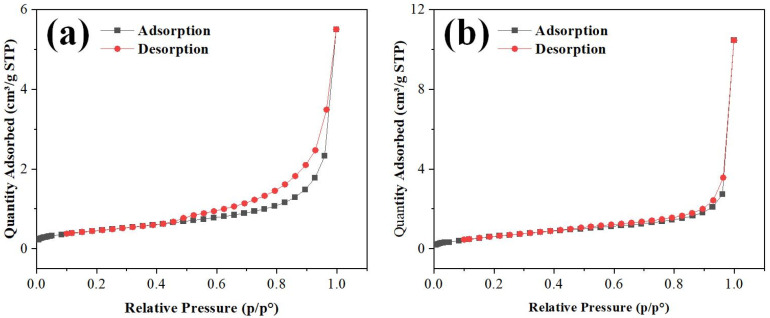
N_2_ adsorption and desorption isotherms of (**a**) ZnO and (**b**) ZnO-Pt/Ru composites.

The adsorption–desorption isotherms of ZnO and ZnO-Pt/Ru in an N_2_ atmosphere were measured using a BET analyzer. Their specific surface areas and pore size distributions were analyzed. As shown in Figure 6a,b, the isotherms of both materials exhibit similar curve shapes, classified as type IV isotherms according to the IUPAC classification. These isotherms exhibit the following characteristics: in the low relative pressure region (p/p^o^ < 0.3), the adsorption quantity remains low and the curve is relatively flat, indicating the predominance of monolayer adsorption. In the medium relative pressure range (p/p^o^ ≈ 0.3–0.8), the adsorption amount gradually increases, suggesting the occurrence of multi-layer adsorption. At high relative pressures (p/p^o^ > 0.8), the adsorption quantity rises sharply, and a distinct hysteresis loop appears near p/p^o^ ≈ 1.0, signifying capillary condensation. The presence of a noticeable hysteresis loop between the adsorption and desorption curves further confirms the existence of a well-defined porous structure in the materials.

By comparing Figure 6a,b, it can be observed that ZnO-Pt/Ru has a higher adsorption capacity than ZnO, suggesting that ZnO-Pt/Ru likely possesses a larger specific surface area and a wider pore size distribution. To further verify this hypothesis, the specific surface areas and pore sizes of both materials were analyzed, yielding values of 1.6777 m^2^/g and 19.782 nm for ZnO, and 2.6451 m^2^/g and 22.584 nm for ZnO-Pt/Ru, respectively. Compared to pure ZnO, ZnO-Pt/Ru demonstrates significant enhancements in both specific surface area and average pore diameter. Therefore, the incorporation of Pt and Ru effectively increases the specific surface area of ZnO, thereby enhancing the interaction between the gas-sensitive material and target gas molecules and improving its adsorption and desorption capabilities.

**Figure 7:**
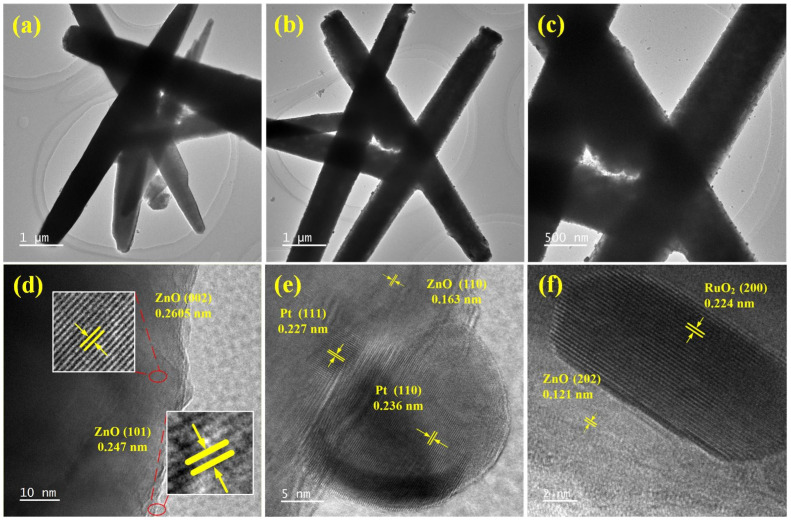
TEM images of (**a**) ZnO, (**b**,**c**) ZnO-Pt/Ru at different magnifications; HRTEM images of (**d**) ZnO, (**e**,**f**) ZnO-Pt/Ru.

Figure 7 shows TEM images of ZnO nanorods and ZnO-Pt/Ru. From Figure 7a–c, it can be seen that the diameter of the original ZnO nanorods ranges from 400 nm to 600 nm. After magnifying the surface of ZnO-Pt/Ru, some particles can be observed randomly distributed on the surface of the ZnO nanorods, indicating that Pt and Ru have been successfully doped on the ZnO nanorod surface. In Figure 7d, the original ZnO nanorods exhibit lattice spacings of 0.247 nm and 0.2605 nm, which belong to the (101) and (002) planes of ZnO, respectively. The lattice spacings measured in Figure 7e were 0.236 nm and 0.227 nm, which belong to the (110) plane and (111) plane of Pt, respectively. Similarly, in Figure 7f, the measured lattice spacing of 0.224 nm was attributed to the (200) plane of RuO_2_.

**Figure 8:**
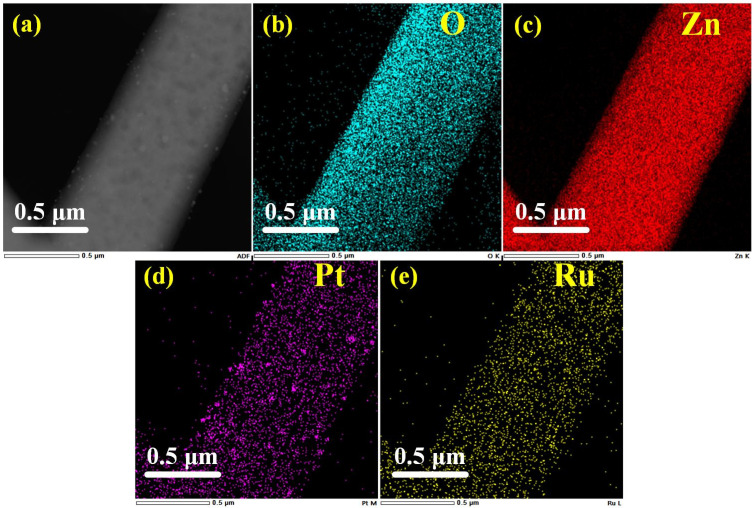
HADDF image of (**a**) ZnO-Pt/Ru and EDS elemental mapping profiles of (**b**) O, (**c**) Zn, (**d**) Pt and (**e**) Ru.

In the HADDF image (Figure 8a), the surface particles can be clearly observed. The EDS spectrum obtained from the HADDF image element mapping is shown in Figure 8b–e, indicating the existence of both Pt and Ru. The elemental composition is approximately Pt 0.36%, Ru 0.1%, Zn 55.05%, and O 44.58%.

### 3.2. Gas-Sensing Performance

**Figure 9:**
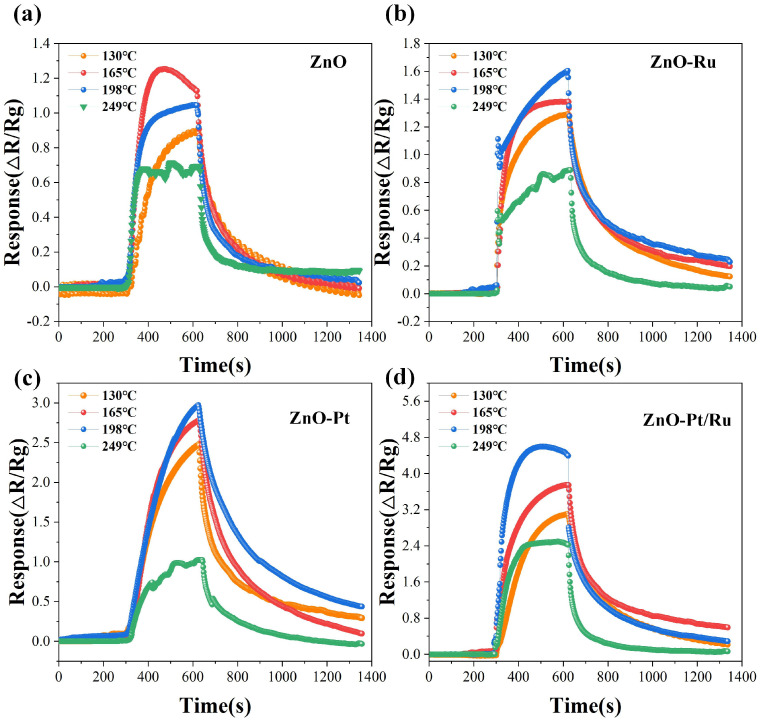
Gas responses of (**a**) ZnO, (**b**) ZnO-Ru, (**c**) ZnO-Pt, and (**d**) ZnO-Pt/Ru sensors to 10 ppm H_2_S at various operating temperatures.

The operating temperature plays a crucial role in the redox reactions involved in the gas adsorption process. It significantly impacts the practical application of sensors. To determine the optimal operating temperature, different voltages were applied to the heating electrodes of the alumina-based sensors, adjusting the corresponding temperatures to 130 °C, 165 °C, 198 °C, and 249 °C. The response of four sensors to 10 ppm H_2_S was measured at these four temperatures (Figure 9a–d). Among all the sensors, the ZnO-Pt/Ru sensor exhibited superior sensitivity across different operating temperatures. Therefore, the optimal operating temperature of ZnO-Pt/Ru was selected as the overall operating temperature. Notably, the ZnO-Pt/Ru sensor demonstrated the highest gas response at 198 °C. Although all four sensors exhibited shorter recovery times at 249 °C, their responses were significantly lower than those at other temperatures. Excessively high operating temperatures may also shorten the sensor’s lifespan. Therefore, 198 °C was selected as the optimum operating temperature.

**Figure 10:**
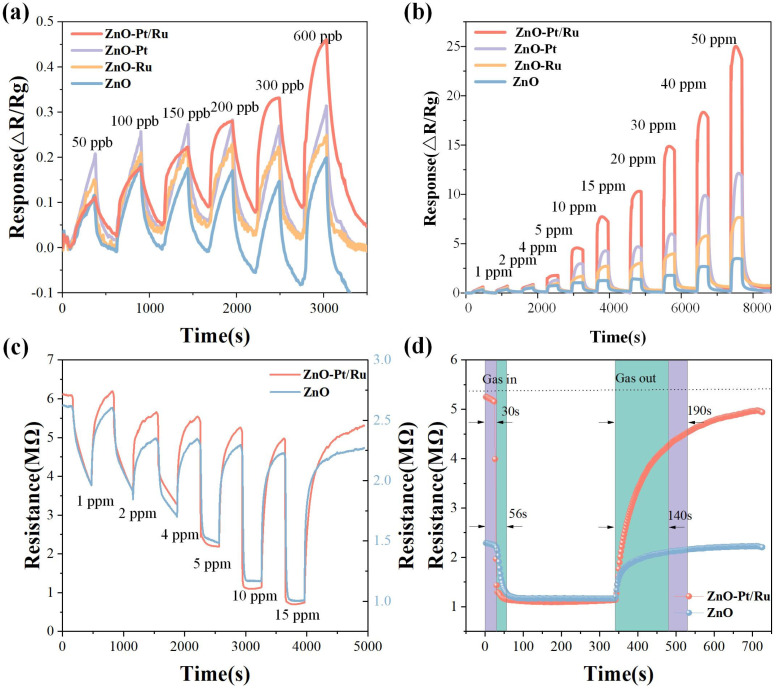
Gas responses of ZnO, ZnO-Ru, ZnO-Pt, and ZnO-Pt/Ru sensors to (**a**) 50–600 ppb H_2_S, (**b**) 1–50 ppm H_2_S at the temperatures of 198 °C, (**c**) dynamic resistance curves of ZnO-Pt/Ru and ZnO sensors with increasing H_2_S concentration at 198 °C, (**d**) response and recovery of ZnO sensors and ZnO-Pt/Ru sensors toward 15 ppm H_2_S.

Figure 10a shows the responses of the four sensors at optimal operating temperatures. At low concentrations, the ZnO-Pt/Ru response increases steadily in proportion to the concentration gradient, while the responses of the other three sensors do not increase significantly. ZnO-Pt/Ru exhibits a 10% response at 50 ppb H_2_S, indicating that ZnO-Pt/Ru has good H_2_S low concentration detection capabilities.

Figure 10b shows the dynamic response of the four sensors at 198 °C under H_2_S concentrations ranging from 1 to 50 ppm. It can be observed that at higher concentrations, the ZnO-Ru sensor exhibited approximately double the response performance than the original ZnO. However, with the increase in test duration and H_2_S gas concentration, the ZnO-Ru response cannot be fully recovered. Additionally, it can be clearly observed that ZnO-Pt/Ru shows a significantly higher response to H_2_S gas than the original ZnO, especially at high concentrations. Under a 50 ppm H_2_S atmosphere, the ZnO-Pt/Ru response was nearly eight times that of the original ZnO (3.5), and the H_2_S response was also better than that of ZnO-Ru and ZnO-Pt. Figure 10c shows the dynamic resistance changes of ZnO-Pt/Ru and original ZnO under exposure to 1, 2, 4, 5, 10, and 15 ppm H_2_S. It can be observed that after exposure to H_2_S gas, the resistance of the gas-sensitive material suddenly increases, reflecting the N-type semiconductor characteristics of ZnO. Moreover, the resistance of ZnO-Pt/Ru increased significantly due to the trace doping of Pt and Ru. In addition, during the desorption of H_2_S, all gas-sensitive materials exhibited varying degrees of the drift phenomenon. The baseline resistance of the original ZnO showed a downward drift, indicating the phenomenon of incomplete recovery. However, although the drift of ZnO-Pt/Ru was also observed, the extent of the drift was significantly reduced compared to the original ZnO.

Figure 10d shows the resistance variation curves of ZnO-Pt/Ru and the original ZnO at a concentration of 15 ppm. The figure demonstrates that while the response intensity of ZnO-Pt/Ru to 15 ppm H_2_S was close to 8, the response speed was still faster than the corresponding original ZnO. In addition, due to its high response intensity, the recovery time of ZnO-Pt/Ru was longer than that of the original ZnO.

**Figure 11:**
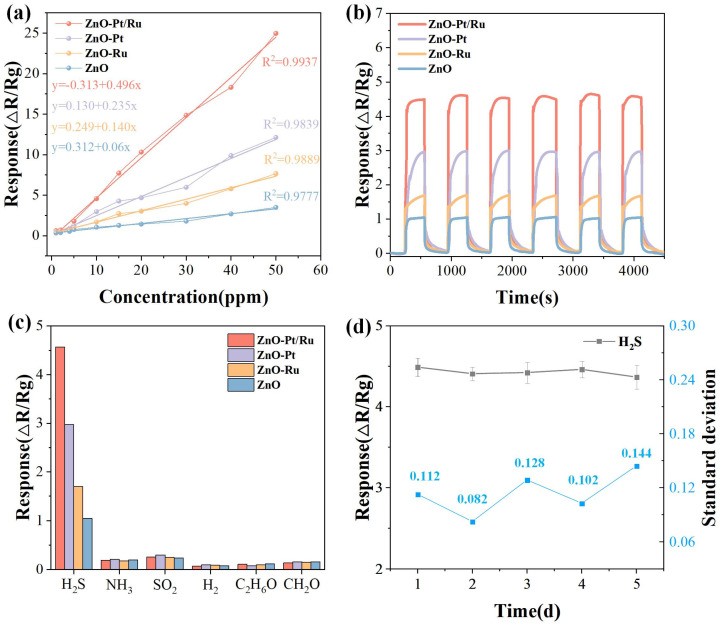
(**a**) Fitting curves as a function of H_2_S concentration in logarithmic coordinates; (**b**) repeatability of the ZnO, ZnO-Ru, ZnO-Pt, and ZnO-Pt/Ru sensors toward 10 ppm H_2_S at 198 °C and (**c**) selectivity of ZnO, ZnO-Ru, ZnO-Pt, and ZnO-Pt/Ru sensors to various target gases; (**d**) response of the ZnO-Pt/Ru sensors to 10 ppm H_2_S over 5 days.

Figure 11a shows the relationship between the responses of the four sensors and H_2_S gas concentration. With the increase in H_2_S concentration, the ZnO-Pt/Ru sensor’s response significantly increased, while the responses of the other sensors increased slowly. Compared to ZnO (R^2^ = 0.9801), ZnO-Ru (R^2^ = 0.9901), and ZnO-Pt (R^2^ = 0.9839), the ZnO-Pt/Ru sensor exhibited a better linear relationship (R^2^ = 0.9944). Its response follows the equation y = −0.313 + 0.496x. Figure 11b shows the dynamic response curves of the four materials to 10 ppm H_2_S at 198 °C. Over six consecutive measurements, all of the sensors exhibited good repeatable dynamic recovery characteristics, indicating that all four materials had excellent repeatability.

The response of the four sensors to 10 ppm of H_2_S, NH_3_, SO_2_, H_2_, C_2_H_6_O, and CH_2_O was tested (Figure 11c). The ZnO-Pt/Ru sensor had the largest response to H_2_S and a very low response to other gases, showing good selectivity.

In order to characterize the stability of the sensor in the experiment, we tested five sets of H_2_S gas response data each day for a period of five days. As shown in Figure 11d, the standard deviation values fluctuated slightly between 0.082 and 0.144. These small variations indicate that the ZnO-Pt/Ru sensor’s response to 10 ppm H_2_S remained basically smooth and did not show significant fluctuations, indicating excellent stability.

The above results indicate that the ZnO-Pt/Ru sensor developed in this study exhibits superior gas-sensing properties to most ZnO-based sensors (Table 1).

### 3.3. Gas-Sensing Mechanism

For N-type MO gas sensors, such as ZnO, the charge carriers were free electrons. Oxygen in the air is adsorbed to the surface of ZnO. Simultaneously, the free electrons in the conduction band of ZnO flow toward the O_2_ molecules adsorbed on the ZnO surface. The O_2_ on the ZnO surface gradually transforms into various chemically adsorbed oxygen species, such as O_2_^−^ and O_2_^2−^ [19,31]. This process can be explained by Equations (1)–(3). At this time, the concentration of charge carriers in the conduction band of ZnO decreases, forming an electron depletion layer, which leads to an increase in the resistance of ZnO. When ZnO is transferred from the air and atmosphere to H_2_S, the adsorbed oxygen on the ZnO surface undergoes a redox reaction with H_2_S, releasing free electrons into the conduction band of ZnO. As a result, the carrier concentration increases, the depletion layer narrows, and the resistance decreases, which was consistent with the results in Figure 10c,d. This process is expressed in Equations (4) and (5):

(1) $$O_{2(g)}\to O_{2(\mathrm{ads})}$$

(2) $$O_{2(\mathrm{ads})}+e^{-}\to O_{2(\mathrm{ads})}^{-}$$

(3) $$O_{2(\mathrm{ads})}^{-}+e^{-}\to {2O}_{\left(\mathrm{ads}\right)}^{-}$$

(4) $$H_{2}S_{\left(g\right)}+O_{2(\mathrm{ads})}^{-}\to H_{2(g)}+{\mathrm{SO}}_{2(g)}+e^{-}$$

(5) $$H_{2}S_{\left(g\right)}+{2O}_{\left(\mathrm{ads}\right)}^{-}\to H_{2(g)}+{\mathrm{SO}}_{2(g)}+{2e}^{-}$$

In addition, another mechanism, known as sulfidation and desulfurization, occurs during the adsorption of H_2_S by ZnO [35,36]. Studies have shown that ZnO undergoes a sulfidation process at temperatures above 150 °C in the presence of H_2_S, as shown in Equations (6)–(8). The resulting ZnS further contributes to a decrease in the resistance of ZnO.

(6) $$H_{2}S_{\left(g\right)}+\mathrm{ZnO}\to H_{2}O+\mathrm{ZnS}$$

(7) $$H_{2}S_{\left(g\right)}+{2O}_{2(\mathrm{ads})}^{-}\to H_{2(g)}+{\mathrm{SO}}_{4(g)}^{-}+e^{-}$$

(8) $$H_{2}S_{\left(g\right)}+\mathrm{ZnO}+{4O}_{\left(\mathrm{ads}\right)}^{-}\to H_{2}O+{\mathrm{ZnSO}}_{4}^{-}+{4e}^{-}$$

Since the desulfurization reaction typically requires temperatures above 300 °C, but the working temperature of the sensors in this study was below 300 °C, the desulfurization process was unlikely to occur under these conditions. This conclusion also explains the phenomenon of resistance drift of ZnO-Pt/Ru from H_2_S to air in Figure 10c.

In this study, the gas-sensing performance of ZnO-Pt/Ru was significantly superior to that of the original ZnO primarily due to the presence of Pt and Ru nanoparticles and their respective sensitization effects. The analysis of XPS peak fitting and TEM crystal plane spacing revealed that the form of Pt in the composite was both elemental Pt and PtO_x_. The work function of Pt (5.65 eV) is greater than that of ZnO (3.30 eV), resulting in the formation of a Schottky junction between Pt and ZnO. A potential barrier exists at the interface between Pt and ZnO, causing free electrons in the conduction band of ZnO to transfer to Pt nanoparticles.

**Figure 12:**
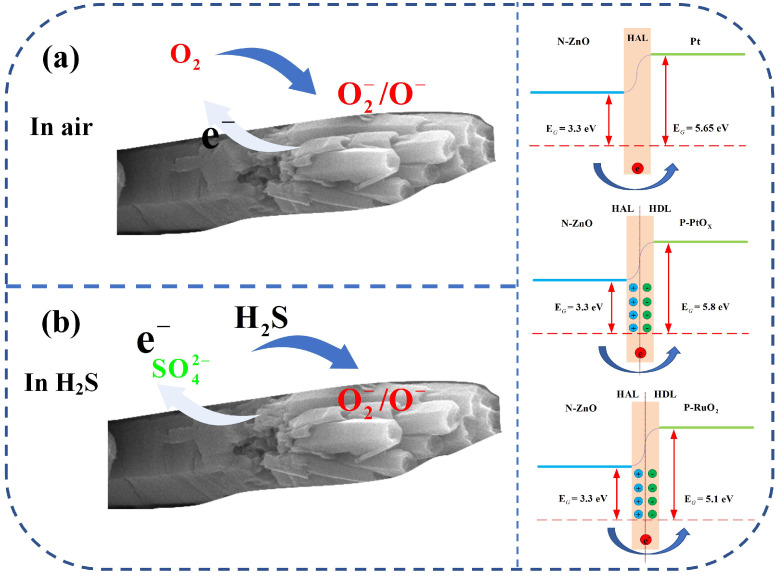
Schematic diagrams of the gas-sensing mechanism for ZnO-Pt/Ru under different gas atmospheres (**a**) O_2_ and (**b**) H_2_S.

As shown in Figure 12, this process increases the space charge area, which effectively thickens the electron depletion layer in ZnO. As a result, the resistance of ZnO will increase further, creating a more obvious resistance change. This principle is also consistent with the observation in Figure 10d, where the resistance of ZnO-Pt/Ru is higher than that of the original ZnO.

RuO_2_ and PtO_x_ were formed during calcination in a muffle furnace. Both are P-type semiconductors. A PN junction is formed between ZnO and RuO_2_ (PtO_x_) due to differences in their work functions [37]. Electrons transfer from ZnO to RuO_2_ (PtO_x_) until their Fermi levels are equal. This process is accompanied by the bending of the energy band at the interface, forming an electron depletion layer at the interface junction and ultimately promoting the thickening of the electron depletion layer [38,39] and enhances gas-sensing performance by promoting the adsorption of chemically adsorbed oxygen. The improved performance of ZnO-Pt/Ru is attributed to the functionalization of Pt and the electronic sensitization mechanism at the PN junction of PtO_x_ and RuO_2_. This mechanism accelerates the movement of charge carriers during the reaction between the target gas and the sensor, significantly enhancing H_2_S sensing performance.

## 4. Conclusions

ZnO nanorods were synthesized on alumina ceramic substrates via a hydrothermal growth method. Subsequently, a certain amount of Pt and Ru were doped into the in situ-grown ZnO nanorods by a drip calcination method, successfully verifying the presence of the doped elements. Compared to the original ZnO and ZnO samples doped with Pt or Ru separately, ZnO-Pt/Ru exhibited a significantly improved response at the optimal operating temperature of 198 °C, which is relatively low compared to conventional ZnO operating temperatures. This improvement reduced sensor energy consumption and extended sensor lifetime. Additionally, ZnO-Pt/Ru showed fast response and good recovery characteristics, with a linear relationship in the 1–50 ppm concentration range, good repeatability, and a high response of nearly 26 at 50 ppm. Moreover, ZnO-Pt/Ru demonstrated excellent selectivity, which was attributed to the electronic sensitization and chemical sensitization mechanisms introduced by doped Pt and Ru, efficiently distinguishing the reducing gas H_2_S.

## Figures and Tables

**Table 1:** Performance comparison of ZnO-Pt/Ru Gas Sensor with Other H_2_S Gas Sensors.

Materials	Temp (°C)	Concentration (ppm)	Response	Detection Limit (ppm)	Response/Recovery Time (s)	Reference
ZnO (nanosheet)	70	100	23 ^a^	NA	252/3697	[30]
ZnO (film)	300	100	3.2 ^a^	5/NA	10/198	[31]
CuO/ZnO	250	25	1.9 ^a^	NA	23/NA	[32]
ZIF-8/ZnO	RT	10	2.08 ^a^	0.05/10	>600/>900	[11]
ZnO/rGO	90	2	1.10 ^a^	2/100	8/32	[33]
ZnO-Al	200	600	12.5 ^a^	NA	90/209	[34]
TGS2602	NA	1	~1.88 ^a^	0.1/3	<25/~20	FIGARO
ZnO-Pt/Ru	198	15	7.9 ^b^	0.05/NA	30/190	This work

^a^ Response is defined as Ra/Rg or Rg/Ra, Ra: resistance of the sensor in air, Rg: resistance of the sensor exposed to target gas. ^b^ Response is defined as ΔR/Ra × 100% or ΔR/Rg × 100%, ΔR: the change in resistance, which equals to |Ra − Rg|.

## Data Availability

Data are contained within the article.

## References

[1] Ali F.I.M., Awwad F., Greish Y.E., Mahmoud S.T. (2019). Hydrogen Sulfide (H_2_S) Gas Sensor: A Review. IEEE Sens. J..

[2] Pandey S.K., Kim K.-H., Tang K.-T. (2012). A review of sensor-based methods for monitoring hydrogen sulfide. Trac-Trends Anal. Chem..

[3] Li W., Shahbazi M., Xing K., Tesfamichael T., Motta N., Qi D.-C. (2022). Highly Sensitive NO_2_ Gas Sensors Based on MoS_2_@MoO_3_ Magnetic Heterostructure. Nanomaterials.

[4] Gao X.H., Krokowski D., Guan B.J., Bederman I., Majumder M., Parisien M., Diatchenko L., Kabil O., Willard B., Banerjee R. (2015). Quantitative H_2_S-mediated protein sulfhydration reveals metabolic reprogramming during the integrated stress response. eLife.

[5] Hsueh T.-J., Wu S.-S. (2021). Highly sensitive Co_3_O_4_ nanoparticles/MEMS NO_2_ gas sensor with the adsorption of the Au nanoparticles. Sens. Actuators B Chem..

[6] Zhang H., Wang Y., Zhu X., Li Y., Cai W. (2019). Bilayer Au nanoparticle-decorated WO_3_ porous thin films: On-chip fabrication and enhanced NO_2_ gas sensing performances with high selectivity. Sens. Actuators B Chem..

[7] Shewale P.S., Patil V.B., Shin S.W., Kim J.H., Uplane M.D. (2013). H_2_S gas sensing properties of nanocrystalline Cu-doped ZnO thin films prepared by advanced spray pyrolysis. Sens. Actuators B Chem..

[8] Xuan J.-Y., Zhao G.-D., Shi X.-B., Geng W., Li H.-Z., Sun M.-L., Jia F.-C., Tan S.-G., Yin G.-C., Liu B. (2021). *In-situ* fabrication of ZnO nanoparticles sensors based on gas-sensing electrode for ppb-level H_2_S detection at room temperature. Chin. Phys. B.

[9] Datta N., Ramgir N., Kaur M., Ganapathi S.K., Debnath A.K., Aswal D.K., Gupta S.K. (2012). Selective H_2_S sensing characteristics of hydrothermally grown ZnO-nanowires network tailored by ultrathin CuO layers. Sens. Actuators B Chem..

[10] Fan C., Sun F., Wang X., Huang Z., Keshvardoostchokami M., Kumar P., Liu B. (2019). Synthesis of ZnO Hierarchical Structures and Their Gas Sensing Properties. Nanomaterials.

[11] Wu X., Xiong S., Gong Y., Gong Y., Wu W., Mao Z., Liu Q., Hu S., Long X. (2019). MOF-SMO hybrids as a H_2_S sensor with superior sensitivity and selectivity. Sens. Actuators B Chem..

[12] Kruefu V., Wisitsoraat A., Tuantranont A., Phanichphant S. (2015). Ultra-sensitive H_2_S sensors based on hydrothermal/impregnation-made Ru-functionalized WO_3_ nanorods. Sens. Actuators B Chem..

[13] Yu A., Li Z., Yi J. (2021). Selective detection of parts-per-billion H_2_S with Pt-decorated ZnO nanorods. Sens. Actuators B Chem..

[14] Su Y., Chen P., Wang P., Ge J., Hu S., Zhao Y., Xie G., Liang W., Song P. (2019). Pd-loaded SnO_2_ hierarchical nanospheres for a high dynamic range H_2_S micro sensor. RSC Adv..

[15] Choi S.-W., Katoch A., Sun G.-J., Kim S.S. (2013). Bimetallic Pd/Pt nanoparticle-functionalized SnO_2_ nanowires for fast response and recovery to NO_2_. Sens. Actuators B Chem..

[16] Chen L., Zhang Y., Sun B., He J., Kang S., Hua Z.-Q., Tian C. (2022). Surface modification of WO_3_ nanoparticles with Pt and Ru for VOCs sensors. Chin. J. Anal. Chem..

[17] Chen X., Shen Y., Zhou P., Zhong X., Li G., Han C., Wei D., Li S. (2019). Bimetallic Au/Pd nanoparticles decorated ZnO nanowires for NO_2_ detection. Sens. Actuators B Chem..

[18] Jiang K., Chen T., Sun J., Quan H., Zhou T. (2023). Pd/Pt-Bimetallic-Nanoparticle-Doped In_2_O_3_ Hollow Microspheres for Rapid and Sensitive H_2_S Sensing at Low Temperature. Nanomaterials.

[19] Duan X., Xu D., Jia W., Sun B., Li R., Yan R., Zhao W. (2024). Pt and black phosphorus co-modified flower-like WS_2_ composites for fast NO_2_ gas detection at low temperature. Nanoscale.

[20] Zhu Z., Huang S.-H., Liu C.-X., Wu R.-J. (2018). Fabrication of Pd-Pt/ZnO for High Sensitive Gaseous Formaldehyde Sensor. J. Nanosci. Nanotechnol..

[21] Liu J., Zhang L., Fan J., Zhu B., Yu J. (2021). Triethylamine gas sensor based on Pt-functionalized hierarchical ZnO microspheres. Sens. Actuators B Chem..

[22] Kruefu V., Inpan U., Leangtanom P., Arkarvipath C., Kongpark P., Phokharatkul D., Wisitsoraat A., Tuantranont A., Phanichphant S. (2018). Enhanced Gas-Sensing Performances of Ru-Loaded p-Type Co_3_O_4_ Nanoparticles. Phys. Status Solidi A-Appl. Mater. Sci..

[23] Xu J., Han J., Zhang Y., Sun Y.A., Xie B. (2008). Studies on alcohol sensing mechanism of ZnO based gas sensors. Sens. Actuators B Chem..

[24] Zhang S., Wang C., Qu F., Liu S., Lin C.-T., Du S., Chen Y., Meng F., Yang M. (2020). ZnO nanoflowers modified with RuO_2_ for enhancing acetone sensing performance. Nanotechnology.

[25] Yang M., Lu J., Wang X., Zhang H., Chen F., Sun J., Yang J., Sun Y., Lu G. (2020). Acetone sensors with high stability to humidity changes based on Ru-doped NiO flower-like microspheres. Sens. Actuators B Chem..

[26] Patil V.L., Vanalakar S.A., Tarwal N.L., Patil A.P., Dongale T.D., Kim J.H., Patil P.S. (2019). Construction of Cu doped ZnO nanorods by chemical method for Low temperature detection of NO_2_ gas. Sens. Actuators A Phys..

[27] Kim B.-Y., Cho J.S., Yoon J.-W., Na C.W., Lee C.-S., Ahn J.H., Kang Y.C., Lee J.-H. (2016). Extremely sensitive ethanol sensor using Pt-doped SnO_2_ hollow nanospheres prepared by Kirkendall diffusion. Sens. Actuators B Chem..

[28] Guo L., Xie N., Wang C., Kou X., Ding M., Zhang H., Sun Y., Song H., Wang Y., Lu G. (2018). Enhanced hydrogen sulfide sensing properties of Pt-functionalized α-Fe_2_O_3_ nanowires prepared by one-step electrospinning. Sens. Actuators B Chem..

[29] Hu K., Li Y., Ge C., Bai L., Liu G., Qiao G., Kang S.G., Kim E.J., Wang M. (2023). Room-temperature ppb-level NO_2_ sensitivity of three-dimensional ordered macroporous Au-loaded SnO_2_ under intermittent UV light irradiation. Sens. Actuators B Chem..

[30] Wang M., Luo Q., Hussain S., Liu G., Qiao G., Kini E.J. (2019). Sharply-precipitated spherical assembly of ZnO nanosheets for low temperature H_2_S gas sensing performances. Mater. Sci. Semicond. Process..

[31] Nimbalkar A.R., Patil M.G. (2017). Synthesis of ZnO thin film by sol-gel spin coating technique for H_2_S gas sensing application. Phys. B-Condens. Matter.

[32] Nadargi D.Y., Tamboli M.S., Patil S.S., Dateer R.B., Mulla I.S., Choi H., Suryavanshi S.S. (2020). Microwave-Epoxide-Assisted Hydrothermal Synthesis of the CuO/ZnO Heterojunction: A Highly Versatile Route to Develop H_2_S Gas Sensors. Acs Omega.

[33] Balasubramani V., Sureshkumar S., Rao T.S., Sridhar T.M. (2019). Impedance Spectroscopy-Based Reduced Graphene Oxide-Incorporated ZnO Composite Sensor for H_2_S Investigations. Acs Omega.

[34] Kolhe P.S., Shinde A.B., Kulkarni S.G., Maiti N., Koinkar P.M., Sonawane K.M. (2018). Gas sensing performance of Al doped ZnO thin film for H_2_S detection. J. Alloys Compd..

[35] Motaung D.E., Mhlongo G.H., Bolokang A.S., Dhonge B.P., Swart H.C., Ray S.S. (2016). Improved sensitivity and selectivity of pristine zinc oxide nanostructures to H_2_S gas: Detailed study on the synthesis reaction time. Appl. Surf. Sci..

[36] Wang D., Chu X., Gong M. (2007). Hydrothermal growth of ZnO nanoscrewdrivers and their gas sensing properties. Nanotechnology.

[37] Wendel P., Periyannan S., Jaegermann W., Klein A. (2020). Polarization dependence of ZnO Schottky barriers revealed by photoelectron spectroscopy. Phys. Rev. Mater..

[38] Jang J.-S., Choi S.-J., Kim S.-J., Hakim M., Kim I.-D. (2016). Rational Design of Highly Porous SnO_2_ Nanotubes Functionalized with Biomimetic Nanocatalysts for Direct Observation of Simulated Diabetes. Adv. Funct. Mater..

[39] Zhang S., Yange M., Liang K., Turak A., Zhang B., Meng D., Wang C., Qu F., Cheng W., Yang M. (2019). An acetone gas sensor based on nanosized Pt-loaded Fe_2_O_3_ nanocubes. Sens. Actuators B Chem..

